# Novel pathomechanistic insights into lysosomal storage disorders: how neuron-intrinsic cGAS-STING signaling drives disease progression

**DOI:** 10.1038/s41392-024-01901-5

**Published:** 2024-08-16

**Authors:** Maximilian Frosch, Marco Prinz

**Affiliations:** 1 Institute of Neuropathology, University of Freiburg, Faculty of Medicine, Freiburg, Germany; 2 Signalling Research Centres BIOSS and CIBSS, University of Freiburg, Freiburg, Germany

**Keywords:** Molecular neuroscience, Neuroimmunology

In their recent paper published in *Nature Cell Biology*,^1^ Wang and colleagues defined a pathogenetic mechanism among a variety of lysosomal storage disorders (LSDs) mediating neuronal death and disease progression. The authors observed neuron-intrinsic activated cGAS-STING signaling triggered by accumulating cytosolic dsDNA, and thus, they provide a valuable target for directed therapies.

LSDs comprise a group of over 70 monogenetic diseases, mostly inherited autosomal recessive. Each is rare, but collectively, they affect approximately 1 in 5000 individuals.^2^ LSDs present as clinically heterogeneous diseases, however, frequently containing a pediatric neurodegenerative component. Disease hallmarks are progressive lysosomal substrate accumulation and lysosomal dysfunction. The underlying genetic mutations and the accumulating substrates are relatively well characterized for most LSDs. In contrast, the detrimental secondary effects caused by accumulations are considerably less understood. In recent years, progress has been made in elucidating the connection between lysosomal dysfunction and neurodegeneration. For example, an impaired autophagy pathway is a common feature in LSDs. Dysfunctional autophagy, in turn, has been shown to cause neurodegeneration by the accumulation of inclusion bodies in neurons.^3^ An interesting finding from an immunological perspective is that microglia recognize accumulating β-glucosylceramide through the Mincle receptor in a mouse model of Gaucher disease (GD), resulting in microglial activation and induction of phagocytosis of living neurons.^4^ However, our understanding of how substrate accumulations or lysosomal dysfunction initiate cellular responses that lead to cell death is still incomplete. Here, Wang et al.^1^ unveiled another connection between lysosomal dysfunction and neurodegeneration, namely via STING signaling, a key mediator of inflammation in the framework of infection, cell stress, and neurodegeneration.^5^

Wang and colleagues first examined whether STING signaling is active in LSDs. Analyzing *Hexb*^*−/−*^ mice, a commonly used model of the GM2-gangliosidosis Sandhoff disease, displaying marked neurodegeneration and innate immune activation, they found an accumulation of STING proteins in cerebellar neurons and the brainstem. Immunoblotting of cerebellar and brainstem lysates consistently revealed a substantial STING signaling activation shown by upregulation of TBK1, IRF3, and pSTAT1. Vice versa, genetic ablation of STING in *Hexb*^*−/−*^ mice, achieved by generating *Hexb*^*−/−*^*Sting1*^*−/−*^ mice, resulted in a significant reduction of ISG gene induction (i.e., *Isg15*, *Ccl5*, *Cxcl10*). But still, it is unclear how STING signaling is activated in *Hexb*^*−/−*^ mice. To test lysosomal dysfunction as potential trigger, the authors introduced sorting nexin protein (SNX)8, that promotes lysosomal tubulation and whose overexpression facilitates lysosomal substrate degradation, specifically into neurons of *Hexb*^*−/−*^ mice via intracerebroventricular injection of an AAV9-based vector. AAV-Snx8 injected mice demonstrate a significant downregulation of STING signaling, exemplified by STING immunohistochemistry in cerebellar Purkinje cells. These observations suggest a constitutive active STING signaling in *Hexb*^*−/−*^ neurons driven by lysosomal dysfunction (Fig. 1).

**Fig. 1:**
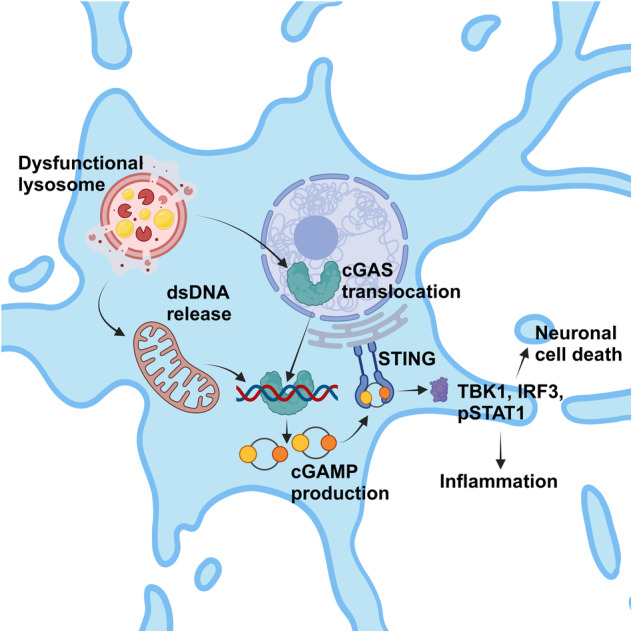
Potential mechanism of cGAS-STING activation in LSDs. Progressive lysosomal substrate accumulation and lysosomal dysfunction are hallmarks in LSDs. In neurons, lysosomal dysfunction triggers cytoplasmic cGAS translocation and dsDNA release from mitochondria. Upon dsDNA binding, cGAMP is synthesized and binds to STING dimers at the endoplasmic reticulum, resulting in the production of TBK1, IRF3, and pSTAT1. Ultimately, cGAS-STING activation leads to neuronal cell death. Figure made by using Biorender

Since *Hexb*^*−/−*^ mice show rapid progressive motor dysfunction and die prematurely, *Hexb*^*−/−*^*Sting1*^*−/−*^ mice represent a valuable tool for investigating the effect of STING inhibition on disease symptoms and survival. Indeed, Wang et al. performed a range of motoric behavioral tests and observed substantial improvements in double-knockout mice. Notably, *Sting1* ablation extended the life span of *Hexb*-deficient mice.

Searching for a molecular basis for these findings, the authors analyzed the brains of *Hexb*^*−/−*^*Sting1*^*−/−*^ mice and found significantly ameliorated microglia activation and astrogliosis. In addition, neuronal loss was significantly reduced in *Hexb*^*−/−*^*Sting1*^*−/−*^ mice. This impact of *Sting1* ablation raises the question of whether these effects are neuron intrinsic or extrinsic. Therefore, Wang and colleagues generated *caMK2α*^*Cre*^*Sting1*^*fl/fl*^*Hexb*^*−/−*^ to disrupt cGAS-STING signaling in excitatory neurons specifically. Interestingly, neuron-specific impaired STING signaling protected against the loss of neurons in *Hexb*^*−/−*^ mice, clearly implicating a neuron-intrinsic role of STING signaling in causing neuronal cell death.

To translate their findings from Sandhoff mice into other LSDs, the authors generated cellular models of CLN10 disease (*Ctsd*), Niemann-Pick disease (*Npc1*), and GD (*Gba*) via CRISPR-mediated knockout in N2a neuronal cells. Again, STING signaling was induced in all cellular models in vitro, shown by increased TBK1, IRF3, and STING and transcriptional upregulation of ISGs. However, these effects were reversible by treating the cells with STING inhibitors H151 or C-176, respectively. In addition, the authors analyzed mouse models of Fabry disease (*Gla*^*−/−*^) and Niemann-Pick disease (*Npc1*^*−/−*^) to show STING activation. Taken together, active STING signaling seems to be a common feature in LSDs.

Lastly, the authors aimed to identify the mechanism by which STING is activated in LSDs. They observed a marked accumulation of cytosolic dsDNA in the brains of *Hexb*^*−/−*^, *Gla*^*−/−*^, and *Npc1*^*−/−*^ mice, mostly co-localized with the mitochondrial marker TOM20. Interestingly, the digestion of cytosolic dsDNA by the introduction of TREX1 into cultured *Hexb*^*−/−*^ embryonic fibroblasts or the elimination of mtDNA in *Hexb*^*−/−*^ N2a cells by treatment with ethidium bromide led to a reduction of STING signaling. Notably, *Hexb*^*−/−*^, *Npc1*^*−/−*^, and *Ctsd*^*−/−*^ N2a cells produce cGAMP, suggesting an active dsDNA-cGAS axis driven by dsDNA from neuronal mitochondria. But how is cytoplasmic translocation of cGAS induced? To test lysosomal dysfunction as trigger, the authors treated HeLa cells and primary neurons with Bafilomycin A1 to induce lysosomal perturbation. Indeed, they observed a nuclear export of cGAS into the cytoplasm, indicating that lysosomal dysfunction is sufficient to induce cGAS cytoplasmic translocation in neurons, which is critical for activating STING signaling in LSDs. To confirm the importance of cGAS in disease progression, *Hexb*^*−/−*^*Ggas*^*−/−*^ and *Gla*^*−/−*^*Cgas*^*−/−*^ double knockouts were generated. Again, in double-knockout mice, STING signaling was reduced, neuronal death was ameliorated, and motor symptoms were attenuated.

Ultimately, Wang et al. provided evidence for the therapeutic potential of targeting cGAS-STING in LSDs. Intracerebroventricular injection of AAV9-mTrex1 viruses in *Hexb*^*−/−*^, *Gla*^*−/−*^, or *Npc1*^*−/−*^ mice led to reduced STING signaling, increased neuronal survival, and improvements in behavioral testing.

In a variety of LSDs, neuronal cell death underlies severe neurological symptoms leading to a devastating disease state. Wang et al. identified a so far unknown pathomechanism by which lysosomal dysfunction causes neuron-intrinsic activation of cGAS-STING signaling leading to neuronal death (Fig. 1). Besides the biological relevance of this study for understanding LSD pathogenesis, their findings are of particular interest for developing LSD therapies that target the convergent downstream cGAS-STING activation. However, since cGAS-STING activation represents a detrimental secondary effect in LSDs, more research is needed to fully elucidate and define primary therapeutic targets preventing substrate accumulation and lysosomal dysfunction in CNS neurons.

## References

[1] Wang, A. et al. Innate immune sensing of lysosomal dysfunction drives multiple lysosomal storage disorders. *Nat. Cell Biol.* **26**, 219–234 (2024). 38253667 10.1038/s41556-023-01339-x

[2] Platt, F. M. et al. Lysosomal storage diseases. *Nat. Rev. Dis. Primers* **4**, 27 (2018). 30275469 10.1038/s41572-018-0025-4

[3] Hara, T. et al. Suppression of basal autophagy in neural cells causes neurodegenerative disease in mice. *Nature* **441**, 885–889 (2006). 16625204 10.1038/nature04724

[4] Shimizu, T. et al. Direct activation of microglia by β-glucosylceramide causes phagocytosis of neurons that exacerbates Gaucher disease. *Immunity* **56**, 307–319. e308 (2023). 36736320 10.1016/j.immuni.2023.01.008

[5] Gulen, M. F. et al. cGAS–STING drives ageing-related inflammation and neurodegeneration. *Nature* **620**, 374–380 (2023). 37532932 10.1038/s41586-023-06373-1 PMC10412454

